# Anti-Melanoma Activity and Potential Mechanism of Purified Potato Protease Inhibitor

**DOI:** 10.3390/foods14061026

**Published:** 2025-03-18

**Authors:** Qiuyan Li, Lu Jiang, Ni Chen, Xingzhi Wang, Jiajun Yao, Zhien Su, Suqing Zhao

**Affiliations:** 1 Department of Pharmaceutical Engineering, School of Biomedical and Pharmaceutical Sciences, Guangdong University of Technology, Guangzhou 510006, China; 13169183397@163.com (Q.L.); chennixx98@163.com (N.C.); wangxingzhi2001@outlook.com (X.W.); 2 Department of Biomedical Engineering, School of Biomedical and Pharmaceutical Sciences, Guangdong University of Technology, Guangzhou 510006, China; 13923960378@163.com (J.Y.); 15625137446@163.com (Z.S.)

**Keywords:** potato protease inhibitor, anti-melanoma activity, antioxidant activity, patatin, isolation and purification

## Abstract

Melanoma is one of the most lethal cancers originating from melanocytes. Its incidence and mortality have been rising rapidly for several decades and have posed a serious threat to human health. Current melanoma treatments are hindered by the scope of application, low efficiency, high cost, and toxic side effects. Due to their affordability and minimal side effects, natural bioactive compounds derived from plants are promising candidates for melanoma treatment. This study aims to delve into the isolation, purification, and characterization of potato proteins and to explore their potential in melanoma treatment. Two potato proteins, patatin PP-1 and aspartate protease inhibitor PP-2, were isolated and purified by a newly developed method in this work, and their physicochemical properties were systematically characterized. Both potato proteins showed great antiproliferative activities and migration inhibition effects on melanoma cells. Meanwhile, Western blotting results illustrated that they could induce endogenous cell apoptosis by regulating the Bax/Bcl-2 pathway. Notably, aspartate protease inhibitor PP-2 demonstrated the best performance in inhibiting the growth and migration of melanoma cells, which might be attributed to the combined effect of its significant antioxidative activity and the inhibition effect of certain necessary protease activities in melanoma. This study provides valuable insights for developing nutraceuticals and therapeutic strategies against melanoma, which can lead to breakthroughs in melanoma treatment.

## 1. Introduction

Melanoma, a highly aggressive cancer originating from melanocytes, is one of the most lethal cancers and the primary cause of death associated with skin cancer [1]. In recent decades, the incidence and mortality of melanoma have kept rising, particularly among males and the elderly groups [2,3]. In the eastern areas of China, the incidence of melanoma also shows a notable rise [4]. Melanoma is mainly treated by surgery, chemotherapy, photodynamic therapy, radiation therapy, etc. [5,6]. However, surgery resection is only suitable for primary melanoma, while other therapy types are hindered by low efficiency, high cost, and toxic side effects [7,8].

An emerging strategy for melanoma treatment is to explore novel natural bioactive compounds derived from plants, due to their great potential, low cost, and minor side effects. Among all bioactive compounds, plant proteinase inhibitors have attracted increasing attention, and their anticancer activities have received particular concern [9]. For example, proteinase inhibitors from cereals (such as millet) have been found to contribute to cancer prevention and inhibit the growth of cancer cells through various mechanisms [10]; proteinase inhibitors lC-pi I and II derived from *Lavatera cashmeriana* have been shown to suppress the growth of human lung cancer cells in vitro [11]; proteinase inhibitors from chickpeas, lupins, common beans, broad beans, and cowpeas have also shown the capability to inhibit the migration of colon cancer cells [12].

Potato proteins are divided into three categories: patatin, proteinase inhibitors, and high-molecular-weight (MW) proteins. Potato proteinase inhibitors (PPIs) account for half of the total potato proteins [13]. Although the total protein amount is relatively low (~2% (*w*/*w*) based on dry weight) [14], the total production of potatoes is considerable [15]. Moreover, a large amount of wastewater containing potato proteins is generated from the process of potato starch production, which often ends up being discarded. Therefore, it is clear that PPIs have wide sources and great potential for utilization [16].

Potato proteinase inhibitors (PPIs) contain a range of inhibitor types. Based on their protein structure categories, they are categorized into serine proteinase inhibitor, aspartic proteinase inhibitor, cysteine proteinase inhibitor, and carboxypeptidase inhibitor [17,18,19]. PPIs have shown excellent radical scavenging activities and promising bioactivities, such as regulating proteolytic activity, inhibiting tumor cell growth, and protecting the immune system [20,21,22,23,24]. However, the anti-melanoma activity of PPIs has not been reported. Meanwhile, current research mostly focuses on the bioactivities of total potato protein, patatin, or a PPI mixture. Systematic studies on purified PPI components are relatively rare, and the relationship between PPI types and functions is mostly unclear.

This work aims to explore and evaluate the anti-melanoma activity of purified potato protease inhibitor PP-2 by isolating and systematically characterizing PP-2, comprehensively measuring its physicochemical properties, assessing its potential to suppress the growth and migration of melanoma cells in vitro, and comparing its anti-melanoma activity with patatin (PP-1). Additionally, the potential mechanisms by which PP-2 exerts inhibitory effects on melanoma cell growth are also explored to provide valuable insights for developing nutraceuticals and therapeutic strategies against melanoma.

## 2. Materials and Methods

### 2.1. Materials and Chemicals

Common yellow heart potatoes were bought from Shangdong, China; B16F0 mouse melanoma cells were purchased from Wuhan Procell Life Science and Technology Co., Ltd., Wuhan, China; HaCaT human immortalized keratinocytes were obtained from BeNa Culture Collection Co., Ltd., in Suzhou, China; anhydrous ethanol was purchased from Comio Chemical Reagent Co., Ltd., in Tianjin, China; methanol was purchased from Tianjin Zhiyuan, China; skim milk powder was purchased from Roles-Bio in Guangzhou, China; SDS lysis buffer and loading buffer were purchased from Beyotime in Shanghai, China; disodium EDTA (EDTA-2Na) was provided by Guangzhou Chemical Reagent Factory, Guangzhou, China; sodium bicarbonate and sodium chloride were obtained from Tianjin Damao Chemical Reagent Factory, Tianjin, China; Dulbecco’s Modified Eagle Medium (DMEM), 0.25% trypsin-EDTA, Roswell Park Memorial Institute (RPMI) 1640 medium, phosphate-buffered saline (PBS), and antibiotics (penicillin and streptomycin) were all purchased from Gibco (Gaithersburg, MD, USA); fetal bovine serum (FBS) was purchased from Biological Industries, Israel; cell proliferation and cytotoxicity assay kits, BCA protein assay kits, Triton X-100, and RNase A solution were all purchased from Biosharp in Beijing, China; 4% paraformaldehyde cell fixative was bought from Shanghai Sun Biotechnology Co., Ltd., Shanghai, China; the Annexin V-FITC/PI apoptosis detection kit was purchased from Hangzhou Lianke Biotechnology Co., Ltd., Hangzhou, China; BeyoECL Moon was purchased from Shanghai Beyotime Biotechnology Co., Ltd., Shanghai, China; 3,4-dihydroxy-L-phenylalanine (L-DOPA), ammonium persulfate, N, N, N’, N’-tetramethylethylenediamine, tris hydroxymethyl aminomethane (Tris), and glycine were all purchased from Shanghai Macklin Biochemical Technology Co., Ltd., Shanghai, China; Tween 20 was purchased from Shanghai Aladdin Biochemical Technology Co., Ltd., Shanghai, China; protein markers were purchased from Thermo Fisher, Waltham, MA, USA. The chemicals employed in this work were analytical- or reagent-grade and were used as received, unless indicated otherwise.

### 2.2. Instruments

The morphologies of the protein samples were captured on a German ZEISS Sigma 300 Scanning Electron Microscope (SEM) (ZEISS, Oberkochen, Germany). The types and amounts of hydrolyzed amino acids in the protein were detected on a UK Biochrom 30+ Amino Acid Analyzer (Biochrom Ltd., Cambridge, UK). Absorbances at specific wavelengths of the samples were recorded on a Multiskan™ FC ELISA reader (Thermo Scientific, Waltham, MA, USA). Clone formation and scratch assays were imaged using an inverted optical microscope from Chongqing Aote Optical, Chongqing, China. Cell cycle and apoptosis were analyzed using an American Agilent flow cytometer. Western blot detection was performed using a chemiluminescence gel imaging system from Bio-Rad (Hercules, CA, USA).

### 2.3. Recovery and Purification of Potato Protein

Potatoes were cleaned, the peel was removed, and the potatoes were cut into small cubes. The same weight of distilled water was added. The mixture was ground using a commercial blender and then quickly immersed in a sodium bisulfite solution (20 mg·mL^−1^) to prevent polyphenol oxidation. The mixture was then kept at 4 °C for 30 min to precipitate the starch. The starch was removed; the solution was gathered and centrifuged at 10,000× *g* for 30 min at 4 °C. The supernatant underwent membrane filtration with a 0.22 μm filter to obtain clear potato juice (PJ), which was used to simulate potato starch industrial wastewater. The potato juice was further dialyzed for 3 days at 4 °C and then freeze-dried to obtain potato total protein. The obtained total protein was loaded onto the Sephadex G-75 column (1.6 × 80 cm) and pre-equilibrated with 25 mM Tris-HCl buffer (pH 7.4). Each fraction was collected into tubes (1 mL per tube). The protein content was measured by BCA to quantify the protein concentration in each test tube, and the protein concentration profile was plotted according to the tube number. The purified potato protein solutions were collected and freeze-dried for further usage.

### 2.4. Sodium Dodecyl Sulfate–Polyacrylamide Gel Electrophoresis (SDS-PAGE)

The purity and MW of the purified potato proteins were visualized by SDS-PAGE. Two purified potato proteins were dissolved in pure water. A total of 40 µL of the protein solution was mixed with 10 µL of 5 × concentrated loading buffer. The mixture underwent thermal treatment at 100 °C for 10 min to denature the proteins. The proteins were then separated by electrophoresis using a polyacrylamide gel system containing 5% stacking gel and 12% separating gel. Then, the gel was stained with Coomassie Brilliant Blue. Finally, the calibration curve was obtained by drawing the log of the MW of standard proteins against their relative migration rates. The purity of the proteins could be visualized through a single stripe, while the MW of unknown proteins could be determined based on their relative migration rate.

### 2.5. BCA Method for Detecting Protein Content

The protein concentration was determined by the BCA Protein Assay Kit. Protein standards (BSA) were diluted to 2, 1, 0.5, 0.25, and 0 mg·mL^−1^. BCA reagent A was mixed with B in a 50:1 volume ratio to prepare the working solution. A total of 200 μL of the working solution was added into each standard sample (25 μL) in a 96-well plate and incubated under 37 °C for 30 min. After incubation, the absorbance of each sample was measured at 562 nm using a microplate reader. The calibration curve was graphed according to the absorbance values of the standard solutions. Subsequently, the concentration of the samples was counted according to the calibration curve.

### 2.6. Solubility Determination of Potato Protein

Protein solutions (5 mg·mL^−1^) were prepared under different pH conditions (3~11) and were agitated at 25 °C for 2 h. The solutions were centrifuged at 10,000 rpm for 15 min. The protein contents in the supernatant were determined by the BCA Protein Assay Kit and the corresponding solubilities were calculated.

### 2.7. Glycoprotein Staining

The characteristic of patatin was demonstrated by the Periodic Acid–Schiff (PAS) method using a Glycoprotein Stain Kit [25]. The sample was diluted to 1 mg·mL^−1^ with 5 × MonoColor protein loading buffer and heated at 95 °C for 5 min. Proteins were separated by SDS-PAGE electrophoresis using the above-mentioned condition; then, the glycoprotein staining of gel was operated. The gel was fixed by 50% methanol for 30 min, rinsed two times with ultrapure water, and treated with an oxidizing agent for 15 min, followed by staining. The glycoproteins were visualized as light pink or magenta bands against a colorless background. The positive control was horseradish peroxidase (containing 16% glycoprotein), and the negative control was soybean trypsin inhibitor (containing no glycoprotein).

### 2.8. Pepsin Inhibition Activity Assay

The pepsin inhibition activity assay was operated using the Lowry method, with bovine serum albumin as the standard protein [26]. The pepsin inhibition activity of the potato protein was measured by the hydrolysis degree of hemoglobin at pH 4.0. A total of 100 μg of potato protein (H_2_O instead of the sample as control) and 100 μg of pepsin were both dissolved in 10 mM acetate buffer (pH 4.0) and placed under 37 °C for 30 min. A total of 150 μg of hemoglobin substrate was introduced and maintained under 37 °C for another 30 min. The remaining activity of pepsin was then determined. The inhibition rate of the enzyme was determined as the percentage of the reduced pepsin activity due to the presence of the inhibitor under a given condition. The inhibition rate of pepsin activity was obtained based on the formula below:

Inhibition rate of pepsin activity (%) = (A_c_ − A_s_)/A_c_ × 100% (1)

where A_c_ is the absorbance of the control, representing original pepsin activity without inhibition; A_s_ is the absorbance of the sample treated with potato protein.

### 2.9. Hydrolyzed Amino Acid Composition of Potato Protein

The purified potato protein sample was weighed, and 4 mL of 1:1 hydrochloric acid (approximately 6 mol·L^−1^) was added. The sample was subjected to nitrogen for 15 min using a nitrogen evaporator and then sealed in a tube. The sealed sample hydrolyzed at 110 °C for 24 h; then, it was cooled and diluted to 100 mL. A 2 mL aliquot of the diluted sample was taken and placed in the nitrogen evaporator at 60 °C until drying. After adding 0.02 mol·L^−1^ HCl, the mixture was vortexed and underwent filtration by a 0.22 µm filter. The composition and mass fraction of 17 hydrolyzed amino acids in the protein sample were investigated by an amino acid automatic analyzer.

### 2.10. Scanning Electron Microscope (SEM) Analysis

The sample was attached to a conductive adhesive and gold-coating was performed for 45 s by a Quorum SC7620 sputter coater. The surface morphology of the sample was then recorded on a ZEISS Sigma 300 Scanning Electron Microscope (SEM).

### 2.11. Antioxidant Activity

#### 2.11.1. Reducing Power Activity

The reducing power of a compound could reflect its antioxidant potential to some extent [27]. The reducing power of the potato protein sample was tested based on a procedure from the literature with some modifications [28,29]. In detail, a series of protein solutions with concentrations of 0.25, 0.5, 1, 2, and 4 mg·mL^−1^ was prepared. Then, 1 mL of each protein solution with different concentration gradients was taken and mixed with 1 mL PBS and 1 mL 1% (*m*/*m*) potassium ferricyanide reagent. The mixture was incubated at 50 °C for 20 min and 10% (*m*/*m*) trichloroacetic acid was added. The mixture was then centrifuged at 4000 rpm for 10 min, and 1 mL of the supernatant was taken and mixed well with 1 mL H_2_O and 1 mL 0.1% (*m*/*m*) FeCl_3_. Then, the absorbance of the mixed solution was measured at 700 nm. PBS was used as the control. The reducing power activity was obtained based on the following formula:

Reducing power (%) = (A_x_ − A_0_)/A_0_ × 100% (2)

where A_0_ is the absorbance of the control; A_x_ is the absorbance of the potato protein solutions.

#### 2.11.2. Hydroxyl Radical Scavenging Activity

This activity was surveyed in view of the Fenton reaction system according to a method from the literature with some modifications [30,31]. Similarly, a series of protein solutions with concentrations of 0.25, 0.5, 1, 2, and 4 mg·mL^−1^ was prepared. In total, 1 mL of each protein solution with different concentration gradients was taken and mixed with 1 mL each of FeSO_4_ (9 mM), salicylic (9 mM), and H_2_O_2_ (8.8 mM) solutions. The mixture was incubated under 37 °C for 20 min and underwent centrifugal separation at 4000 rpm for 10 min. The absorbance of the supernatant was recorded at 510 nm. Distilled water and H_2_O_2_ solutions served as the blank and control, respectively. The hydroxyl radical scavenging activity was determined based on the formulae below:

Hydroxyl radicals scavenging activity (%) = [A_c_ − (A_s_ − A_b_)]/A_c_ × 100% (3)

where A_c_ is the absorbance of the control; A_s_ is the absorbance of the potato protein samples; A_b_ is the absorbance of the blank.

#### 2.11.3. DPPH Radicals Scavenging Activity

This activity was tested based on a reported procedure with some modifications [32,33,34,35]. Similarly, a series of protein solutions with concentrations of 0.25, 0.5, 1, 2, and 4 mg·mL^−1^ were prepared. A total of 2 mL of each protein solution with different concentration gradients was taken and mixed with 2 mL DPPH solution (0.02 g·L^−1^, absolute ethanol as the solvent). The mixture was incubated at 25 °C in the dark for 30 min and absorbance at 517 nm was measured. Absolute ethanol and DPPH solution were recognized as the blank and control, respectively. The DPPH radical scavenging activity was determined based on the formula below:

Radicals scavenging activity = [A_c_ − (A_s_ − A_b_)]/A_c_ × 100% (4)

where A_c_ is the absorbance of the control; A_s_ is the absorbance of the potato protein samples; A_b_ is the absorbance of the blank.

The three types of antioxidant activities mentioned above were assessed by plotting scatter plots of relative activity (%) versus sample concentration. The exponential trendlines of the plots were calculated, and the corresponding equations were used to predict the IC_50_ values, which are the concentrations with a 50% inhibition rate of relative activity.

### 2.12. Cell Culture

HaCaT cells were cultured in DMEM high-glucose medium containing 10% fetal bovine serum (FBS) and 1% penicillin–streptomycin (P/S). B16F0 cells were cultured in RPMI-1640 medium supplemented with 15% FBS and 1% P/S (all *v*/*v*). All cells were cultured at 37 °C, with 5% CO_2_ concentration and 95% relative humidity.

### 2.13. Cell Viability Assay

B16F0 cells were seeded in 96-well plates (1.0 × 10^4^ cells/well) and cultured at 37 °C with 5% CO_2_ for 24 h to allow for cell adhesion. Next, the cells were cultured for 24 h in fresh medium containing different concentrations of potato proteins (0, 0.25, 0.5, 1, 2, or 4 mg·mL^−1^; 0 mg·mL^−1^ as the control). Then, 10% CCK-8 was added under dark conditions and incubated for 1.5 h. Pure 10% CCK-8 medium solution without cells and the administered sample was used as the blank. The absorbance of each well at 450 nm was measured using a microplate reader. B16F0 cell viability was calculated by the following formula:

(5) $$\mathrm{Cell}\;\mathrm{viability}\;(\%)=\frac{A-A_{b}}{A_{c}-A_{b}}\times \;100\%$$

A is the absorbance value of the well with the administered sample (cell + medium + sample + CCK-8), A_b_ is the absorbance value of the blank (medium + CCK-8), and Ac is the absorbance value of the well without the administered sample.

Cell viability studies of protein samples to HaCaT cells were conducted using a similar method.

### 2.14. Melanin Content Assay

B16F0 cells were seeded at a density of 1.0 × 10^5^ cells/well in 6-well plates and incubated at 37 °C with 5% CO_2_ for 48 h with or without proteins (0, 0.25, 0.5, 1, 2, or 4 mg·mL^−1^; 0 mg·mL^−1^ as the control). After incubation, the cells were washed twice with PBS and collected after centrifugating at 1200 rpm, for 3 min at room temperature. The cells were lysed in 1 M NaOH [10% (*v*/*v*) DMSO] at 80 °C for 1 h, and the melanin content was measured by spectrophotometry at 405 nm. Pure 1 M NaOH [10% (*v*/*v*) DMSO] medium solution without cells and the administered sample was used as the blank. The melanin contents in the control wells were set as 100%, and the melanin contents in other wells were recorded as the percentage of the control wells. The relative melanin content was determined based on the formula below:

(6) $$\mathrm{Relative}\;\mathrm{melanin}\;\mathrm{content}\;(\%)=\frac{A_{t}-A_{0}}{A_{c}-A_{0}}\times \;100\%$$

where A_t_ is the absorbance of the well with the administered sample (cell + medium + sample + 1 M NaOH [10% (*v*/*v*) DMSO]), A_0_ is the absorbance of the blank (medium + 1 M NaOH [10% (*v*/*v*) DMSO]), and A_c_ is the absorbance of the well without the administered sample (cell + medium + 1 M NaOH [10% (*v*/*v*) DMSO]).

### 2.15. Cell Tyrosinase Activity Assay

Tyrosinase activity was estimated through evaluating the oxidative degree of L-DOPA. B16F0 cells were inoculated at 5.0 × 10^3^ cells/well in 96-well plates and incubated under 37 °C and 5% CO_2_ for 24 h. The cells were then cultured for 48 h in fresh medium containing varying concentrations of potato proteins (0, 0.25, 0.5, 1, 2, or 4 mg·mL^−1^; 0 mg·mL^−1^ as the control). The cultured cells were washed with cold PBS, PBS with 0.1% (*v*/*v*) Triton X-100 was added in, and the cells were subsequently lysed at -80 °C for 30 min. A total of 150 μL of the 0.1% L-DOPA PBS solution was mixed with the lysed cells. Cells were incubated at 37 °C for 1 h, and absorbances were recorded at 475 nm. The relative cell tyrosinase activity was determined as follows:

(7) $$\mathrm{Cell}\;\mathrm{tyrosinase}\;\mathrm{activity}\;(\%)=\frac{A_{t}}{A_{0}}\times \;100\%$$

A_t_ is the absorbance value of the well with the administered sample, and A_0_ is the absorbance value of the well without the administered sample.

### 2.16. Colony Formation Assay

The cells were seeded at 1 × 10^3^ cells/well in a 6-well plate. A medium with varying concentrations of proteins (0, 0.25, 0.5, 1, 2, or 4 mg·mL^−1^; 0 mg·mL^−1^ as the control) was added into each well. The plate was incubated under 37 °C with 5% CO_2_ for 7–8 days until visible colonies were formed. Methanol was added to fix the colonies for 20 min, then 3 drops of Giemsa stain reagent I were added. The treated cells were incubated for 1 min. Later, 6 drops of reagent II were immediately added and mixed thoroughly for 8 min. Then, reagents I and II were discarded, and the distilled water was used to wash cells thrice. After Giemsa staining, colonies appeared blue-purple, while the surrounding area of the plate was colorless. The number of the formed cell colonies was observed and recorded by images. Grayscale values of images were calculated by imageJ version 1.53e software to provide more reliable results compared with the colony counting method.

### 2.17. Cell Wound Scratch Assay

The cells were seeded at 1.0 × 10^6^ cells/well in a 6-well plate. When the cell confluence reached more than 95%, a serum-free medium containing varying concentrations of proteins (0, 0.125 or 0.25; 0 mg·mL^−1^ as the control) was added. The cell monolayer surface was scratched by the tip until a blank area was formed in the cell layer and then incubated at 37 °C with 5% CO_2_ for 24 h. The scratch area was observed and recorded at different time points with a microscope. The cell coverage area and the healing rate were determined by comparing the coverage areas of the treated group with the control group with the help of ImageJ software.

### 2.18. Cell Cycle Analysis

B16F0 cells were cultured at 1 × 10^5^ cells/well in a 6-well plate until logarithmic growth phase and incubated at 37 °C with 5% CO_2_ and saturation humidity. After 24 h, the supernatant was discarded, and protein media of varying concentrations (0, 1, 2, or 4 mg·mL^−1^; 0 mg·mL^−1^ as the control) were added for further cultivation. After 48 h, the cells were collected and washed once with PBS. Next, 75% ethanol was cooled, and 1 mL was added, gently mixed, and fixed overnight at 4 °C. Then, the cells were centrifuged, and the supernatant was discarded. Cells were further treated with 400 μL RNAse solution under 37 °C for 30 min. After adding 40 μL of PI (propidium iodide) staining solution, cells were incubated for another 30 min at 4 °C in the dark. Finally, the cells were filtered through a 200-mesh filter, and the cell cycle phases were analyzed by a flow cytometer and NovoExpress 1.6.1.0 software.

### 2.19. Apoptosis Analysis

The incidence of apoptosis was detected using an Agilent flow cytometer with an Annexin V-FITC/PI apoptosis detection kit. In detail, cells were cultured in an incubator with the media containing different concentrations of protein (0, 1, 2, or 4 mg·mL^−1^; 0 mg·mL^−1^ as control) for 48 h. After treatment, cells were collected by centrifugation at 800× *g* for 5 min, washed twice with cold PBS, and resuspended in the 1 × binding buffer from the kit. The cells were then incubated with 10 μL of propidium iodide (PI) and 5 μL of Annexin V-FITC solution at room temperature in the dark for 5 min. Samples were analyzed using a flow cytometer, and scatter plots were generated using the NovoExpress software. The total number of apoptotic cells (early and late apoptotic cells) was counted and expressed as the percentage of total cell numbers.

### 2.20. Western Blotting

B16F0 cells were cultured to the logarithmic growth phase at a density of 1 × 10^5^ cells per well in a 6-well plate and treated by protein media following the same procedure on Section 2.18. After 48 h, when the cell confluence reached more than 95%, the cells were washed with PBS and lysed on ice for 10 min with RIPA cell lysis buffer. The protein concentration of the supernatant was determined by the BCA protein assay kit. Proteins (20 µg/lane) were separated by 10% SDS-PAGE and transferred to a nitrocellulose membrane using the semi-dry blotting method. The membrane was blocked with TBST (1 L TBS with 0.5 mL Tween 20) containing 5% (*v*/*v*) nonfat milk on a horizontal shaker at 80 rpm at 25 °C for 2 h and then incubated overnight at 4 °C with the primary antibodies of the target proteins Bcl-2 (1:500), Bax (1:2000), and Caspase-3 (1:2000) and the control protein β-tubulin (1:5000). After the removal of primary antibodies, the membrane was washed three times with 1 × TBS (6.05 g Tris and 85.0 g NaCl made up to 1 L with H_2_O). The membrane was then incubated for 2 h at 25 °C with HRP-conjugated goat anti-rabbit IgG (1:8000) or HRP-conjugated goat anti-mouse IgG (1:8000) in 0.05% (*v*/*v*) Tween-20 in TBST. The membranes were developed using ECL A and ECL B mixed solutions from BeyoELC Moon, and the excess solution was removed before membrane imaging by the ChemiDoc XRS + system. ImageJ software was used to measure the grayscale values of the bands, and the expression level of the target protein was normalized as the gray value of the target protein/control protein. The control group was the untreated B16F0 cells.

### 2.21. Statistical Analysis

Data were recorded as the mean ± standard deviation (SD). Each experiment was performed at least three times in parallel, and differences between groups were compared by analysis of variance (GraphPad Prism 8 software). A significance level of *p* < 0.05 was considered.

## 3. Results and Discussion

### 3.1. Purification of Protein Components from Potato Juice

**Figure 1:**
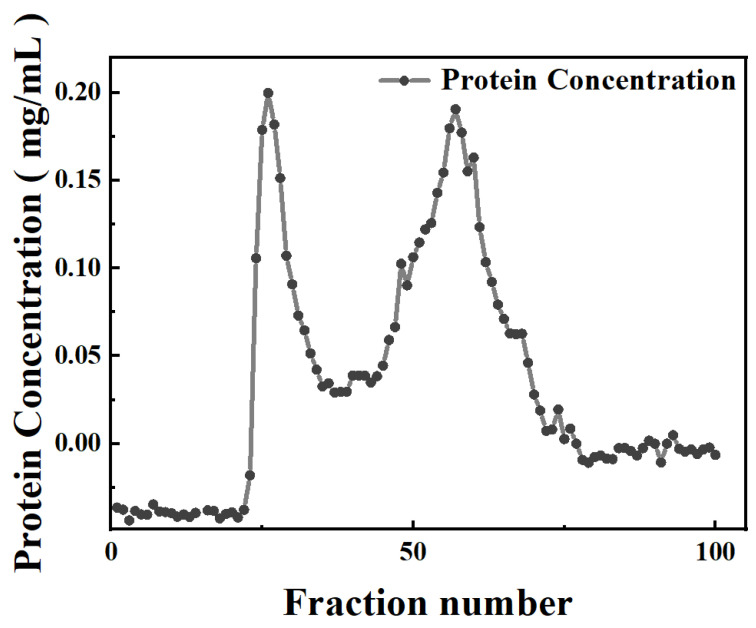
Protein concentrations of eluent fractions purified by Sephadex G-75 column chromatography.

Potatoes were firstly pretreated and blended with the sodium bisulfite (20 mg·mL^−1^) solution. The obtained mixture was then allowed to stand, centrifuged, and filtered to remove starch, resulting in a potato juice that simulated potato starch industrial wastewater. Total protein was obtained from potato juice through dialysis and freeze-drying, with a yield of 3.9% and purity of 89%. The purity of the obtained total protein was slightly higher than the purity of protein obtained by the acid–heat (74.09%), isoelectric point (84.5%), and ultrafiltration (82.7%) methods [36,37]. The total protein was further separated and purified using a Sephadex G-75 column, where proteins were separated based on MW differences. Each eluent fraction separated by the column was collected in tubes, and the protein concentration of each tube was determined by the BCA method (Figure 1). According to the protein concentration profile of eluent fractions, two peak fractions, named PP-1 and PP-2, were collected, with purification yields of 31.65% and 7.9%, respectively. Their purity was verified by SDS-PAGE as a single electrophoretic band. The extraction and purification approach used in this work was mild, low-cost, and relatively convenient. Compared with reported acid–heat flocculation, chemical extraction, and expanded bed adsorption (EBA) chromatography methods, the current method could effectively avoid protein denaturation, minimize protein loss, and avoid the usage of advanced equipment [38,39,40,41].

### 3.2. Homogeneity and Molecular Weight

**Figure 2:**
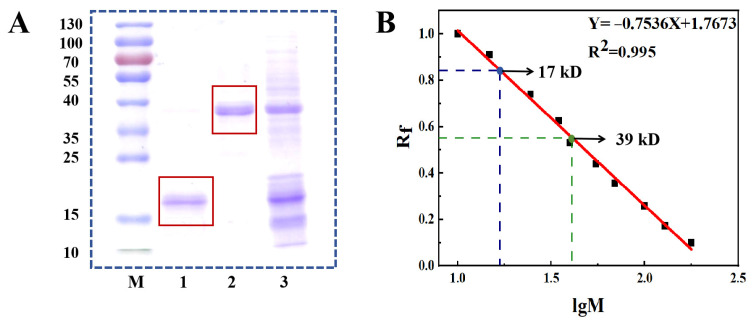
(**A**) SDS-PAGE analysis profile. M: MW marking; Lane 1: PP-2 (Marked with a red box); Lane 2: PP-1 (Marked with a red box); Lane 3: crude total protein fraction; (**B**) standard curve of relative molecular weight of proteins (Blue marks 17 kD, green marks 39 kD).

The homogeneity of the total potato protein and purified protein components PP-1 and PP-2 were visualized on gel by SDS-PAGE electrophoresis (Figure 2A). It can be seen that the total potato protein contains all three reported types of components, high-MW proteins (over 45 kDa), patatins (40–45 kDa), and potato protease inhibitors (PPls, 5–25 kDa). The single stripes on the gel of PP-1 and PP-2 demonstrated their homogeneity and purity. Meanwhile, the MW of PP-1 and PP-2 were 39 kD and 17 kD, respectively, calculated based on the relative MW standard curve of proteins (Figure 2B).

### 3.3. Solubility

**Figure 3:**
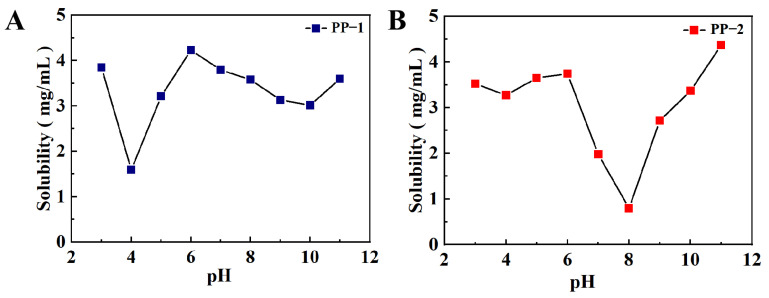
pH solubility curves of purified potato protein PP-1 (**A**) and PP-2 (**B**).

Solubility is a fundamental property of protein. The solubility of both purified proteins exhibited a pH dependence (Figure 3). The solubility of PP-1 reached its lowest point at pH 4 (Figure 3A), while the solubility of PP-2 was lowest at pH 8 (Figure 3B). The pH-dependent solubility profiles are similar to the isoelectric points reported for potato patatin and potato protease inhibitors [42,43].

### 3.4. Protein Type Determination

**Figure 4:**
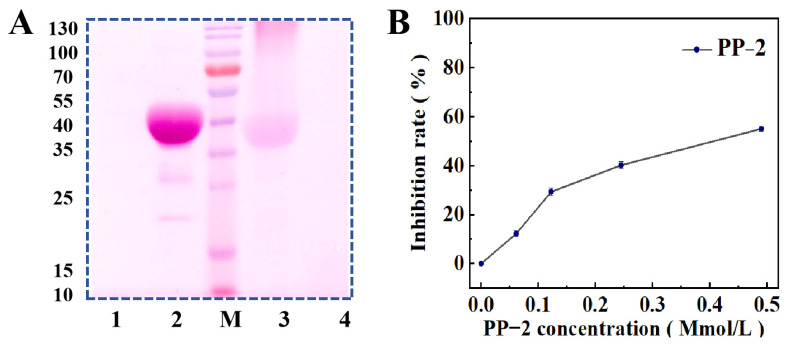
(**A**) SDS-PAGE analysis profile of glycoprotein staining. Lane 1: negative control; Lane 2: positive control; M: MW markers; Lane 3: purified PP-1; Lane 4: purified PP-2; (**B**) inhibition activity of PP-2 on pepsin at different concentrations.

To determine the protein types of PP-1 and PP-2, glycoprotein staining and an enzyme inhibition activity assay were operated. As shown in Figure 4A, PP-1 showed an obvious magenta band after staining, whereas PP-2 had no color display, indicating that PP-1 was a patatin but PP-2 was not. The MW of PP-2 is 17 kDa, which is within the range of potato protease inhibitors. To figure out what kind of protease inhibitor PP-2 is, protease inhibition activity assays were performed. Firstly, its trypsin (a kind of typical serine protease inhibitor) inhibition activity was determined, but the results revealed that it had no inhibitory effect on trypsin. Then, its pepsin inhibition activity was tested. As shown in Figure 4B, PP-2 showed a concentration-dependent pepsin inhibition activity, but the concentration of PP-2 was not linearly related to the inhibition rate. The results demonstrated that PP-2 belonged to the aspartic protease inhibitor family.

### 3.5. Amino Acid Composition

The amino acid composition of the purified PP-1 and PP-2 is listed in Table 1. Essential amino acids in PP-1 accounted for 41.09% of the total amino acids, with leucine having the highest content, followed by lysine, valine, phenylalanine, threonine, methionine, and isoleucine. The results were consistent with the previously reported potato patatin [44]. In PP-2, essential amino acids made up 33.79% of the total amino acids, with valine having the highest content, followed by lysine, leucine, methionine, phenylalanine, threonine, and isoleucine. The amino acid compositions of proteins have been shown to affect their antioxidant activities [45]. Specific amino acids, like ones containing sulfhydryl groups (such as methionine and cysteine) or aromatic rings (such as tryptophan, tyrosine, and phenylalanine), have been found to contain a hydrogen atom, which can react with free radicals, generating antioxidant activity [46,47,48]. According to the data in Table 1, PP-1 contained 9.87% cysteine, 3.20% methionine, 6.07% tyrosine, and 5.59% phenylalanine; while PP-2 contained 32.13% cysteine, 4.04% methionine, 5.30% tyrosine, and 3.93% phenylalanine, respectively. A large number of sulfhydryl groups and aromatic rings were present in the structures of PP-1 and PP-2 and could promote interactions with free radicals, indicating their antioxidant potential.

### 3.6. Microscopic Structures

**Figure 5:**
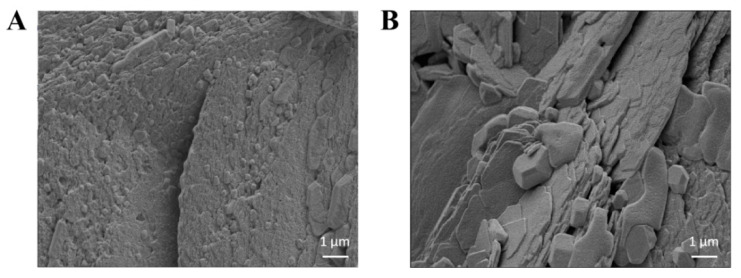
SEM images of potato proteins PP-1 (**A**) and PP-2 (**B**).

The microscopic structures of PP-1 and PP-2 were investigated using SEM (Figure 5). PP-1 and PP-2 showed significantly different microstructures. PP-1 displayed a condensed and aggregated spherical structure on its surface, while PP-2 showed a fragmented, sheet-like, and loose structure. The structure difference might be attributed to the varying protein types. PP-1 contained more polysaccharide or lipid components, which might lead to its smoother surface microstructure [49].

### 3.7. In Vitro Antioxidant Activity

The antioxidant activities of purified proteins, including reducing power, hydroxyl radicals, and DPPH scavenging activities, were tested. Highly efficient antioxidant ascorbic acid (Vc) and ovalbumin (OVA) were used as positive control groups.

**Figure 6:**
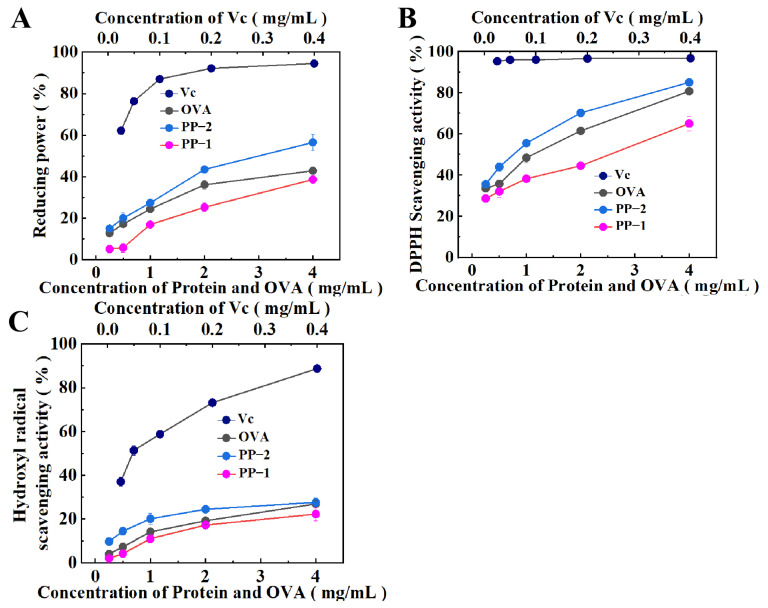
The reducing power (**A**), scavenging activity against DPPH (**B**), and hydroxyl radical scavenging activity (**C**) of PP-1 and PP-2 at different concentrations. Error bars refer to standard deviations.

Reducing power is a measure of the electron-donating ability of a compound, and it is generally positively correlated with its in vitro antioxidant activity. As shown in Figure 6A, PP-1 and PP-2 showed good and concentration-dependent reducing power against Fe^3+^. Although the reducing power of both potato proteins was weaker than Vc, PP-2 showed better performance than OVA, while PP-1 was slightly weaker than OVA.

Compared with the study by Liu et al., the reducing power of the potato proteinase inhibitor (PPI) mixture was superior to that of PP-1 but inferior to that of PP-2 [44]. This indicated that both the PPIs mixture and the purified single-component PPI exhibited greater reducing power than patatin. Moreover, PP-2 showed a stronger reducing power than the PPI mixture, indicating that different PPI components might have varying antioxidant activities, which could be attributed to differences in their contents, their amino acid composition (particularly specific amino acids containing sulfhydryl groups or aromatic rings), and their structural and conformational characteristics.

The DPPH reaction system is a typical radical for assessing the free radical-scavenging ability of samples. The two purified potato proteins exhibited a clear dose–response DPPH scavenging ability (Figure 6B). Within the tested concentration range (0.025~4 mg·mL^−1^), PP-2 showed consistently better DPPH scavenging ability than OVA and comparable DPPH scavenging ability to that of Vc at higher concentrations, while PP-1 exhibited a relatively weak DPPH scavenging ability. The patatin reported by Sun et al. exhibited 75% DPPH free radical scavenging activity at a concentration of 40 mg·mL^−1^ [50], while the patatin (PP-1) in this study showed 65% corresponding activity at a concentration of only 4 mg·mL^−1^. Meanwhile, according to a report by Liu et al., the IC_50_ value of the DPPH radical scavenging activity of the reported PPI mixture was 3.81 mg·mL^−1^ [44], while the IC_50_ value of PP-1 and PP-2 was only 1.97 ± 0.30 and 0.64 ± 0.04 mg·mL^−1^, respectively. These results indicated PP-1 and PP-2 had better DPPH free radical scavenging activities compared to the reported potato proteins.

The ability of the two potato proteins to scavenge the most active hydroxyl radicals was also tested. Both PP-1 and PP-2 showed relatively weak hydroxyl radical scavenging ability, and their hydroxyl radical scavenging ability increased slowly with the increase in concentration (Figure 6C). Within the tested concentration range (0.025~4 mg·mL^−1^), PP-2 showed slightly better hydroxyl radical scavenging ability than OVA. Compared with the reported PPIs, which exhibited an approximately 20% hydroxyl radical scavenging ability at a concentration of 5 mg·mL^−1^ [44], PP-1 and PP-2 showed slightly better hydroxyl radical scavenging ability, 23.5% and 27.6%, respectively, at a concentration of 4 mg·mL^−1^. The relatively low hydroxyl radical scavenging ability might be related to the presence of ethanol in the detection system, which might affect the solubility of the proteins, thereby limiting their binding efficiency with hydroxyl radicals [51].

To more clearly reflect the reducing power and free radical scavenging ability of the tested proteins, half-reducing/scavenging concentrations (IC_50_ values) of oxidant/radicals were calculated and listed in Table 2. By comparing the IC_50_ values, PP-2 exhibited the best DPPH radical scavenging activity and reducing power among proteins and a slightly weaker hydroxyl radical scavenging ability than OVA at higher concentrations. PP-1 overall showed the weakest antioxidant property. The great antioxidant property of PP-2 was probably due to the high contents of amino acids with thiol groups or aromatic rings, especially significantly high contents of cysteine (32.13%). Previous studies showed that bioactive compounds with good antioxidant activities possessed anticancer activity. Inspired by their excellent antioxidant activities [22], it was speculated that both PP-1 and PP-2 had anticancer potential, and the anticancer effect of PP-2 was stronger than that of PP-1.

### 3.8. Cell Cytotoxicity Study

**Figure 7:**
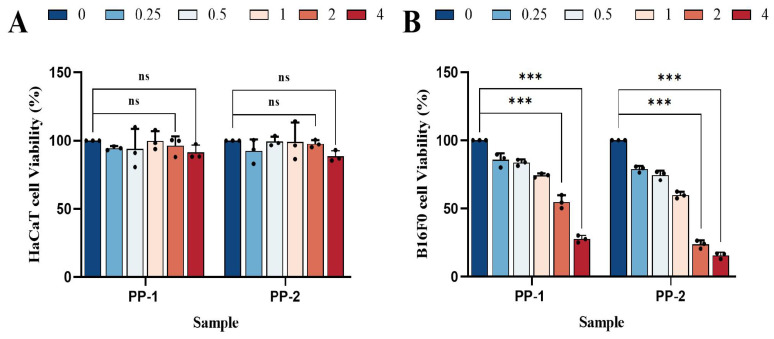
In vitro cytotoxic activity of PP-1 and PP-2 on (**A**) HaCaT and (**B**) B16F0 cells, with concentrations expressed in mg·mL^−1^ (*n* = 3, ns: *p* > 0.05, *** *p* < 0.001).

To assess their potential cytotoxicity and feasibility for pharmaceutical applications, the CCK-8 assay was used to evaluate the effects of PP-1 and PP-2 on the cell viability of HaCaT skin cells and melanoma B16F0 cells. For HaCaT cells, both PP-1 and PP-2 had almost no negative effect on cell viability within a concentration range of 0.25~4 mg·mL^−^^1^. The cell viability of PP-1 and PP-2 could reach 91.3% and 88.5% at the high concentration of 4 mg·mL^−^^1^ (Figure 7A) [52,53]. The high cell viability of HaCaT cells indicated that both PP-1 and PP-2 had great biocompatibility with normal skin cells. In contrast, both PP-1 and PP-2 showed strong anti-proliferative effects on B16F0 cells with a dose-dependent response (Figure 7B). At concentrations of 0.25, 0.5, 1, 2, and 4 mg·mL^−^^1^, the cell viability of PP-1 on B16F0 cell proliferation was 85.6%, 83.6%, 74.4%, 54.9%, and 27.7%, respectively, while the cell viability of PP-2 was 79.0%, 74.5%, 59.9%, 23.8%, and 15.5%, respectively. PP-2 showed better anti-proliferative effects on B16F0 cells than PP-1. Compared with the reported potato patatin, which inhibited the proliferation of mouse melanoma B16 cells by 68% at a concentration of 20 mg·mL^−^^1^ [50], PP-1 was found to inhibit the proliferation of mouse melanoma B16F0 cells by 72.3% at a much lower concentration of 4 mg·mL^−^^1^. Compared with the reported PPI mixture, which inhibited gastrointestinal stromal tumors cells (GIST882) at an IC_50_ of 10.53 mg·mL^−^^1^ [23], PP-2 was observed to inhibit the proliferation of mouse melanoma B16F0 cells by 76.2% at a concentration of 2 mg·mL^−^^1^. The results suggested that PP-1 and PP-2 had significant cytotoxicity on melanoma B16F0 cells, while they were non-irritating to skin cells, which makes them potential anticancer drugs with lower side effects.

### 3.9. Measurement of Cellular Melanin Content

**Figure 8:**
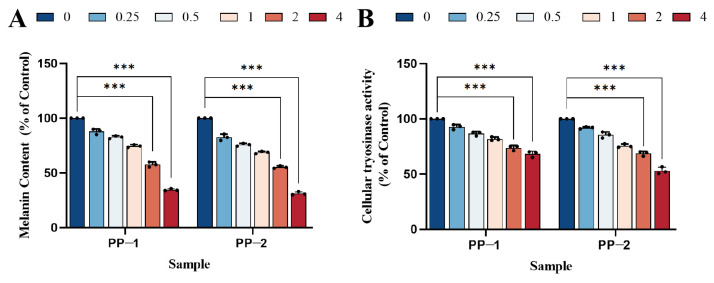
(**A**) Effect of PP-1 and PP-2 on melanin content in B16F0 cells and (**B**) inhibition of tyrosinase activity by PP-1 and PP-2 in B16F0 cells, with concentrations expressed in mg·mL^−1^ (*n* = 3, *** *p* < 0.001).

Melanoma is a malignant tumor arising from the transformation of melanocytes in the skin and other organs. To evaluate whether the inhibition of melanoma cell proliferation was related to melanin content, we measured the melanin levels in B16F0 cells treated with PP-1 and PP-2. Within concentrations between 0.25 and 4 mg·mL^−1^, both potato proteins reduced melanin production in the cells (Figure 8A). Moreover, the melanin content in B16F0 cells treated with PP-2 was lower than that in cells treated with PP-1, especially at higher concentrations. For example, at a concentration of 4 mg·mL^−1^, the melanin content in B16F0 cells was reduced to 34.6% by PP-1, while it was reduced to 31.2% by PP-2. This suggested that PP-2 had a better inhibition effect on melanin production.

### 3.10. Cellular Tyrosinase Inhibition Study

Tyrosinase is a key enzyme that regulates melanin production in cells. To further explore the mechanism of melanin reduction in B16F0 cells, the tyrosinase inhibition effect of potato proteins was studied at a range of concentrations (0, 0.25, 0.5, 1, 2, and 4 mg·mL^−1^). Both proteins exhibited increased inhibition of tyrosinase activity in B16F0 cells as the concentration increased (Figure 8B). At concentrations of 2 and 4 mg·mL^−1^, the tyrosinase activity of PP-1 was reduced to 73.67% and 68.2%, respectively. In comparison, PP-2 exhibited better tyrosinase inhibitory activity, with reductions to 68.5% and 53.0% at the same concentrations. The better tyrosinase inhibition of PP-2 might also be due to its better antioxidant activity, which could indirectly inhibit tyrosinase activity by scavenging free radicals [23]. The results showed that PP-1 and PP-2 might reduce melanin production through the inhibition of tyrosinase activity, which is consistent with previous reports [54].

### 3.11. Cloning Experiment

**Figure 9:**
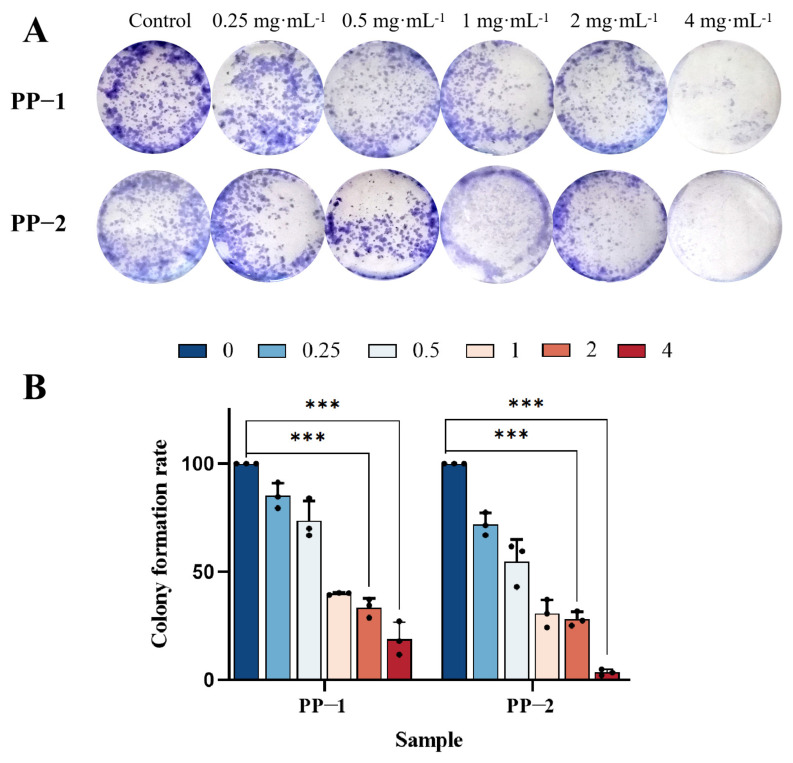
(**A**) Colony formation images of B16F0 melanoma cells treated with PP-1 and PP-2; (**B**) statistical analysis of colony formation rate, with concentrations expressed in mg·mL^−1^ (*n* = 3, *** *p* < 0.001).

The colony formation assay was used to reflect important characteristics of cell proliferation by evaluating two important traits: cell adhesion ability and proliferation ability. B16F0 cells were treated with different concentrations of potato protein (0.25, 0.5, 1, 2 or 4 mg·mL^−1^) for 7–8 days, and differences in colony formation were observed. The colony images showed that the colony formation ability of B16F0 cells was significantly reduced by PP-1 and PP-2 in a dosage-dependent manner. Not only was the number of colonies reduced, but the diameter of individual colonies was also smaller, and Giemsa staining showed a lighter color, with the best effect observed at a concentration of 4 mg·mL^−1^ (Figure 9A). Based on the statistical analysis of colony formation rates, at a concentration of 4 mg·mL^−1^, PP-1 and PP-2 reduced the colony formation rate of B16F0 cells to 18.93% and 3.45%, respectively, indicating a stronger inhibitory effect for PP-2 (Figure 9B).

### 3.12. Cell Wound Scratch Assay

**Figure 10:**
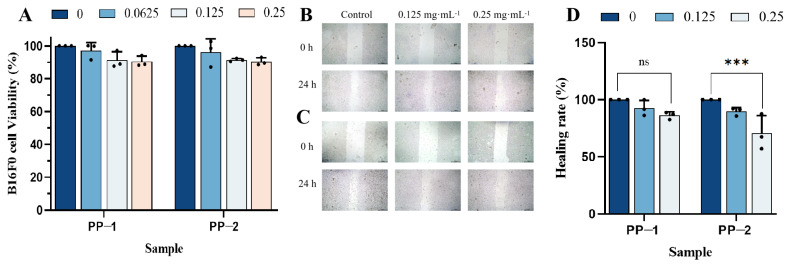
(**A**) In vitro cytotoxic activity of PP-1 and PP-2 on B16F0 cells with a serum-free culture medium. Migration images of B16F0 melanoma cells treated with PP-1 (**B**) and PP-2 (**C**). (**D**) Statistical analysis of scratch wound healing rate, with concentration units in mg·mL^−1^ (*n* = 3, ns: *p* > 0.05, *** *p* < 0.001).

The scratch assay is commonly used to simulate the in vivo migration process of cells. In this experiment, the scratch assay was used to investigate the effect of the two potato proteins on the invasive and metastatic capabilities of adherently growing B16F0 cells. To avoid the effect of potato protein cytotoxicity on their cell migration inhibition performance, a serum-free antiproliferation assay was firstly conducted on B16F0 cells with PP-1 and PP-2 concentrations of 0.0625, 0.125, and 0.25 mg·mL^−1^. With these concentrations, the cell viabilities of PP-1 and PP-2 on B16F0 cell proliferation were all > 90%, indicating that PP-1 and PP-2 at a concentration lower than 0.25 mg·mL^−1^ had no significant cytotoxicity on B16F0 cells (Figure 10A). Based on this result, a wound scratch assay was performed using PP-1 and PP-2 at concentrations of 0.125 and 0.25 mg·mL^−1^. Photos were taken at 0 h and 24 h to record the dynamic changes in “wound” healing (Figure 10B,C). The control group showed more comprehensive and faster healing, while the healing rate of the protein-treated groups gradually slowed down as the concentration increased. After being treated with PP-1 and PP-2 at a concentration of 0.25 mg·mL^−1^ for 24 h, the healing rate of B16F0 was 86.13% and 70.60%, respectively (Figure 10D). Both proteins exhibited certain inhibitory effects on the migration of B16F0 cells, and PP-2 had a better inhibition effect on cell migration.

### 3.13. Cell Cycle

**Figure 11:**
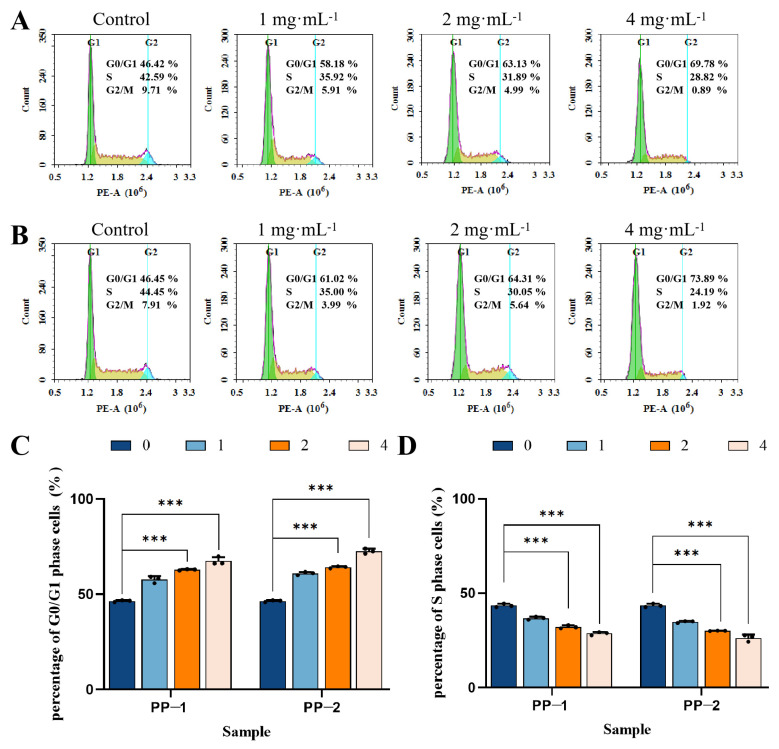
The effect of PP-1 (**A**) and PP-2 (**B**) on the cell cycle distribution of B16F0 cells (The yellow part in the picture represents the S period). Percentages of G0/G1-phase cells (**C**) and S-phase cells (**D**) treated by PP-1 and PP-2, with concentrations expressed in mg·mL^−1^ (*n* = 3, *** *p* < 0.001).

Flow cytometry was used to study the cell cycle distribution of B16F0 melanoma cells to further elucidate the inhibitory effect of the two proteins on cell growth. The number of B16F0 cells treated with the two proteins significantly increased in the G1 phase (Figure 11). When PP-1 was used at concentrations of 0, 1, 2, or 4 mg·mL^−1^, the relative percentage of G1 phase cells increased from 46.42% to 58.18%, 63.13%, and 69.78%, respectively, while the relative percentage of S phase cells decreased from 42.59% to 35.92%, 31.89%, and 28.82%, respectively (Figure 11A). When PP-2 was used at same concentrations, the relative percentage of G1 phase cells increased from 46.45% to 61.02%, 64.31%, and 73.89%, respectively, and the relative percentage of S phase cells decreased from 44.45% to 35.00%, 30.05%, and 24.19%, respectively (Figure 11B). The results indicated that both PP-1 and PP-2 could induce cell cycle arrest in the G1 phase and prevent the cells from entering the S phase, thereby inhibiting DNA synthesis and further cell division. The antiproliferative effect was best at a concentration of 4 mg·mL^−1^, and the effect of PP-2 was better than that of PP-1 (Figure 11C). Compared to the reported patatin, which induced cell cycle arrest in the G1 phase with a proportion of 76.5% at a high concentration of 40 mg·mL^−1^, PP-1 exhibited a similar effect at a much lower concentration of 4 mg·mL^−1^ [50].

### 3.14. Flow Cytometry Detection of Cell Apoptosis

**Figure 12:**
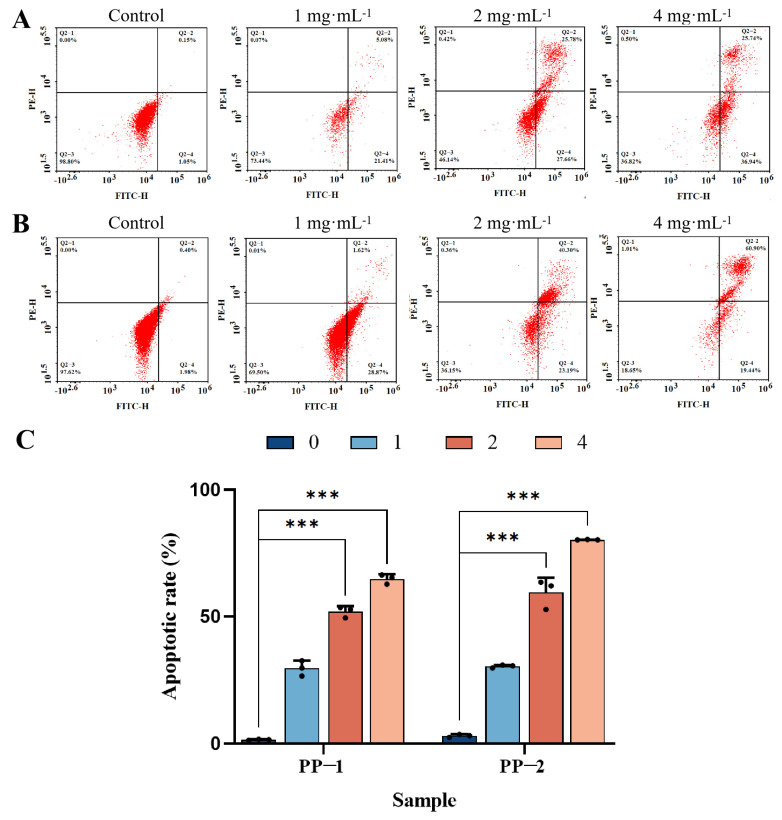
Representative dot plot of flow cytometry analysis of cells treated with different concentrations of PP-1 (**A**) and PP-2 (**B**) (cells were double-stained with Annexin V-FITC/propidium iodide (PI) after 24 h treatment). Q2-1 represents the percentage of necrotic cells; Q2-2 represents the percentage of late-apoptotic cells, Q2-3 represents the percentage of live cells, and Q2-4 represents the percentage of early-apoptotic cells. (**C**) Statistical analysis of cell apoptotic rate, with concentrations expressed in mg·mL^−1^ (*n* = 3, *** *p* < 0.001).

Apoptosis is one of the mechanisms that suppress cell growth. Flow cytometry analysis was conducted to evaluate whether the cell growth inhibition caused by potato proteins was due to apoptosis [55]. Topical dot plots of cell apoptosis analysis for the control and protein-treated groups are shown in Figure 12. For the control group, the percentages of late- and early-apoptotic cells were 0.15% and 1.05%, respectively, while the percentages of live cells and necrotic cells were 98.80% and 0.00%, respectively. For the protein-treated groups, it can be seen that both proteins reduced the proportion of live cells and increased the proportion of apoptotic cells, and the B16F0 cell apoptosis rate significantly increased with the increase in protein concentrations. In detail, after treatment with PP-1 at 4 mg·mL^−1^, the proportion of viable cells decreased from 98.80% in the control group to 36.82%, and the proportions of early- and late-apoptotic cells increased from 1.05% and 0.15% to 36.94% and 25.74%, respectively (Figure 12A). Compared with the reported potato patatin [50], which lowered the proportion of viable cells from 97.7% in the control group to 74.0% and increased the proportion of early- and late-apoptotic cells from 2.1% and 2.5% to 15.1% and 9.1%, respectively, at a concentration of 20 mg·mL^−1^, PP-1 presented a more pronounced cell apoptosis effect on melanoma cells at a lower concentration. Moreover, PP-2 showed a more significant cell apoptosis effect than PP-1; after PP-2 treatment at 4 mg·mL^−1^, the proportion of viable cells decreased from 97.62% in the control group to 18.65%, and the proportions of early- and late-apoptotic cells increased from 1.98% and 0.40% to 19.44% and 60.90%, respectively (Figure 12B). These results indicated that the significant anti-proliferative effects of PP-1 and PP-2 on B16F0 cells were mainly achieved through the induction of apoptosis (Figure 12C).

### 3.15. Western Blotting

**Figure 13:**
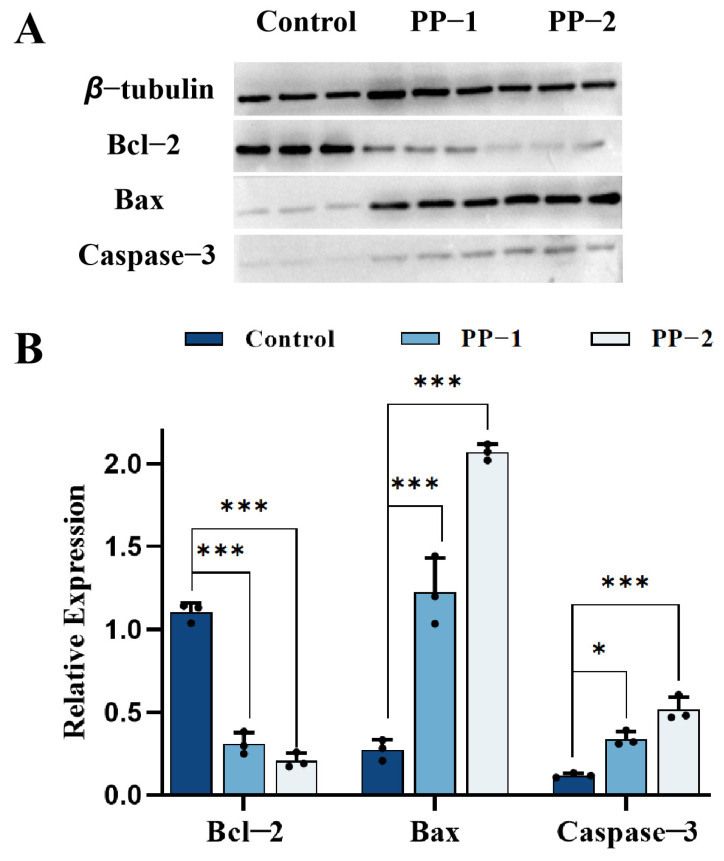
(**A**) Western blot images and (**B**) relative expression bar charts of Bax, Bcl-2, Caspase-3, and β-tubulin in B16F0 cells after treatment with 4 mg·mL^−1^ of PP-1 and PP-2. (*n* = 3, * *p* < 0.05, *** *p* < 0.001).

The inhibition mechanism of potato proteins against B16F0 cells was studied by detecting changes in the expression level of Bcl-2, Bax, and Caspase-3 using Western blot (Figure 13). Since 4 mg·mL^−1^ of potato proteins gave the best B16F0 cell inhibition effect, the concentration of 4 mg·mL^−1^ was chosen for the Western blotting assay. B16F0 cells were treated with 4 mg·mL^−1^ of PP-1 and PP-2 for 24 h. Proteins were collected and subjected to Western blot, and the expression level of the housekeeping protein β-tubulin was used as the reference. After treatment with PP-1 and PP-2, the expression of Bax was enhanced, while the expression of Bcl-2 was reduced in B16F0 cells, corresponding to an increased mitochondrial outer membrane permeability, enhanced mitochondrial pathway apoptosis, and weakening apoptosis inhibition [56]. Meanwhile, the expression of the apoptosis execution protein, Caspase-3, was also increased. The results further demonstrated that PP-1 and PP-2 could induce endogenous cell apoptosis by regulating the Bax/Bcl-2 pathway [57]. Moreover, PP-2 showed a more obvious effect on the increased expression of Bax and Caspase-3 and the reduced expression of Bcl-2, which was consistent with its stronger growth inhibition performance in melanoma cells.

According to previous reports, antioxidants could potentially prevent and treat diseases associated with reactive oxygen species (ROS), including some forms of cancer [58,59]. Meanwhile, protease inhibitors have also been reported to induce apoptosis by affecting the degradation of various short-lived proteins within the cell [60]. Therefore, the enhanced anticancer effect of PP-2 was probably due to the synergistic effect of its significant antioxidant activity and inhibition effect of certain necessary protease activities in melanoma, making it a highly promising anti-melanoma component.

## 4. Conclusions

In summary, two potato protein components were successfully isolated and identified, one patatin (PP-1) and one aspartate protease inhibitor (PP-2). Their homogeneity, molecular weight, composition, and antioxidant properties were comprehensively investigated. Moreover, their potential for the antiproliferation of melanoma cells was systematically explored. Both proteins showed great antiproliferative activities and migration inhibition effects on melanoma cells. It is worth highlighting that the aspartate protease inhibitor PP-2 exhibited more significant antioxidant activities and proliferation inhibition effects on melanoma cells than PP-1. Additionally, a further Western blot study illustrated that potato proteins could induce endogenous cell apoptosis by regulating the Bax/Bcl-2 pathway. This work contributes to a fundamental understanding of purified potato proteins and also highlights PP-2, an aspartic protease inhibitor, as a potential therapeutic agent for melanoma treatment. This study underscores the importance of exploring novel bioactive compounds from traditional food crops to address contemporary health challenges. Future work will focus on the study of the anti-melanoma activity of PP-2 in animal models.

## Figures and Tables

**Table 1:** Amino acid composition of PP-1 and PP-2 (%).

Amino Acid	Content (%)	
	PP-1	PP-2
Thr	5.14	2.73
Val	6.00	9.88
Met	3.20	4.04
Ile	3.17	1.96
Leu	8.08	4.25
Phe	5.59	3.93
His	2.54	1.86
Lys	7.37	5.14
EAAs/TAAs	41.09	33.79
Asp	9.61	6.96
Ser	4.69	2.80
Glu	10.72	5.27
Gly	4.78	3.80
Ala	5.13	2.95
Tyr	6.07	5.30
Arg	3.86	2.38
Pro	4.18	4.63
Cys	9.87	32.13
NEAAs/TAAs	58.91	66.21

EAAs: total essential amino acids; NEAAs: total non-essential amino acids; TAAs: total amino acids.

**Table 2:** IC_50_ values of PP-1, PP-2, OVA, and Vc.

Antioxidant Activity	IC_50_ * (mg·mL^−1^)			
	Vc	OVA	PP-1	PP-2
Reducing power	---	7.74 ± 0.67	12.63 ± 2.62	3.13 ± 0.28
Hydroxyl radical scavenging ability	0.05 ± 0.01	69.99 ± 2.27	209.50 ± 5.90	104.43 ± 2.67
DPPH radical scavenging ability	---	0.89 ± 0.05	1.97 ± 0.30	0.64 ± 0.04

* The IC_50_ values are the averages of three measurements, and ± represents the standard deviation.

## Data Availability

Data will be made available on request.
